# Alternatively spliced MAP4 isoforms have key roles in maintaining microtubule organization and skeletal muscle function

**DOI:** 10.1016/j.isci.2024.111104

**Published:** 2024-10-05

**Authors:** Lathan Lucas, Larissa Nitschke, Brandon Nguyen, James A. Loehr, George G. Rodney, Thomas A. Cooper

**Affiliations:** 1 Chemical, Physical, Structural Biology Graduate Program, Baylor College of Medicine, Houston, TX, USA; 2 Department of Pathology & Immunology, Baylor College of Medicine, Houston, TX, USA; 3 Department of Molecular Physiology and Biophysics, Baylor College of Medicine, Houston, TX, USA; 4 Department of Molecular and Cellular Biology, Baylor College of Medicine, Houston, TX, USA

**Keywords:** Cellular physiology, Molecular physiology, Molecular Structure, Functional aspects of cell biology

## Abstract

Skeletal muscle cells (myofibers) are elongated non-mitotic, multinucleated syncytia that have adapted a microtubule lattice. Microtubule-associated proteins (MAPs) play roles in regulating microtubule architecture. The most abundant MAP in skeletal muscle is MAP4. MAP4 consists of a ubiquitous MAP4 isoform (uMAP4), expressed in most tissues, and a striated-muscle-specific alternatively spliced isoform (mMAP4) that includes a 3,180-nucleotide exon (exon 8). To determine the role of mMAP4 in skeletal muscle, we generated mice that lack mMAP4 and express only uMAP4 due to genomic deletion of exon 8. We demonstrate that loss of mMAP4 leads to disorganized microtubule architecture and intrinsic loss of force generation. We show that mMAP4 exhibits enhanced association with microtubules compared to uMAP4 and that both the loss of mMAP4 and the concomitant gain of uMAP4 cause loss of muscle function. These results demonstrate the critical role for balanced expression of mMAP4 and uMAP4 for skeletal muscle homeostasis.

## Highlights

• Loss of mMAP4 in skeletal muscle results in skeletal muscle weakness
• Loss of mMAP4 in muscle myofibers results in microtubule disorganization
• mMAP4 shows increased microtubule co-localization compared to uMAP4
• Balanced expression of mMAP4 and uMAP4 is required for muscle homeostasis

## Graphical abstract

**Figure:**
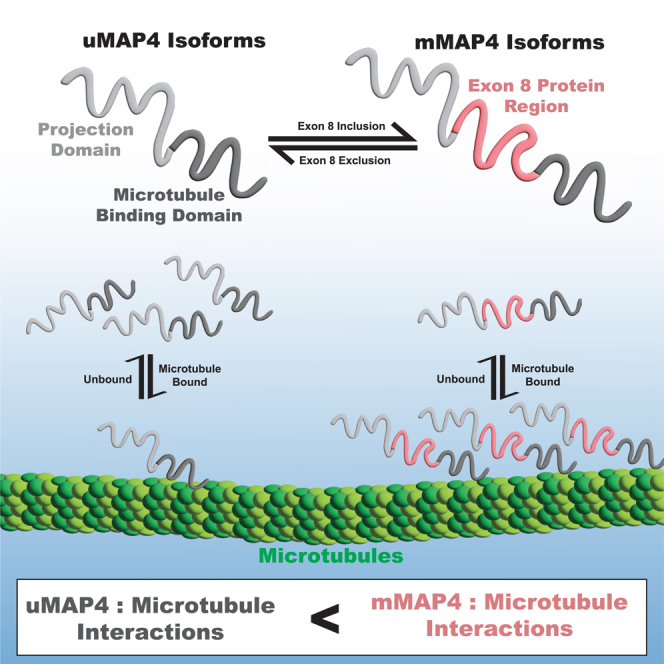

## Introduction

Skeletal muscle cells (myofibers) are a multinucleated syncytium that can be up to 100 μm in diameter and several centimeters in length.^1^ In addition to the components of the contractile apparatus (sarcomere), myofibers require a complex cytoskeleton to provide plasticity during repeated contractions.^2^ Microtubules have evolved to support myofiber development with established importance in myofiber function and homeostasis.^3^^,^^4^^,^^5^^,^^6^^,^^7^ Myofiber microtubules form a dynamic lattice of longitudinal and transverse networks that are tightly regulated by microtubule-associated proteins (MAPs).^3^^,^^8^^,^^9^^,^^10^^,^^11^ The most abundant MAP in myofibers is MAP4, a member of the MAP2/Tau family that share homology in the C-terminal microtubule-binding domain and are largely intrinsically disordered.^12^^,^^13^^,^^14^

**Figure 1:**
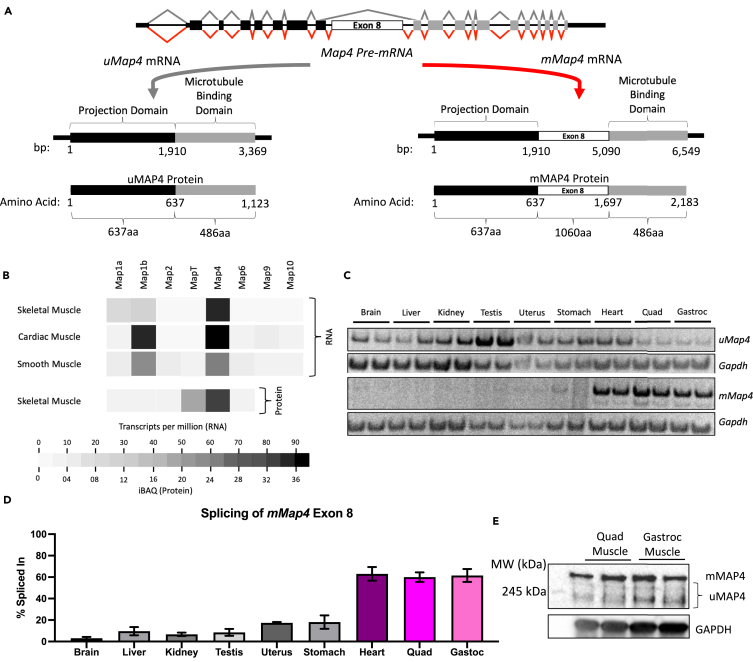
The Map4 transcript undergoes alternative splicing to yield a striated muscle-specific mMAP4 (A) Schematic representation of the Map4 pre-mRNA and the two major alternatively spliced mRNA transcripts produced in skeletal muscle. (B) Heatmap showing the abundance of common MAP mRNAs in striated muscle and proteins in skeletal muscle. (C) Representative RT-PCR (2 replicates per tissue) to quantify the proportions of uMap4 and mMap4 mRNAs expressed in mouse tissues. (D) Percent spliced in of the muscle-specific exon (exon 8) detected by RT-PCR. *n* = 3 mice. Error Bars = SEM. (E) Representative western blot (2 replicates per genotype) of adult mouse quadricep and gastrocnemius skeletal muscles using an antibody to an epitope common to all MAP4 isoforms (Anti-MAP4 SG-10).

MAP4 undergoes alternative splicing to generate a ubiquitously expressed MAP4 isoform (uMAP4) and a striated muscle-specific MAP4 isoform (mMAP4) by inclusion of a 3,180 nucleotide exon (exon 8) to insert 1060 amino acids in-frame upstream of the microtubule binding domain (Figure 1A).^15^^,^^16^ Striated muscle-specific inclusion of exon 8 increases during postnatal development and the exon and its regulation are conserved across many species including human and mice (Figure S1A).^17^ Functional analyses of MAP4 have focused on the uMAP4 isoform that has been shown to play important roles in stabilizing microtubules, regulating mitosis, and modulating kinesin and dynein motor activities.^18^^,^^19^^,^^20^ The mMAP4 isoform was shown to be required for efficient myoblast fusion during differentiation in cell cultures, but that expression of mMAP4 in culture is not sufficient for organizing microtubule networks.^16^^,^^21^ All previous mMAP4 studies were performed in cell culture and the function of mMAP4 in myofibers within skeletal muscle tissue is unknown.

To investigate the role of mMAP4 in skeletal muscle structure and function, we characterized the expression of MAP4 mRNA and protein in mouse tissues and used CRISPR/Cas9 to remove exon 8 from the mouse genome to generate mice that lack the mMAP4 isoform *(Map4*^ΔE8/ΔE8^). *Map4*^ΔE8/ΔE8^ mice had reduced muscle force both *in vivo* and *ex vivo*, without loss of muscle mass, indicative of an intrinsic defect of muscle function. The mice also displayed a significantly disrupted microtubule lattice within isolated myofibers. We show that mMAP4 is functionally distinct from uMAP4 having stronger association with microtubules. We also show that exclusive expression of uMAP4 has a detrimental effect in skeletal muscle. Our results provide an understanding of microtubule regulation in skeletal muscle myofibers while identifying the critical need for mMAP4, balanced with uMAP4, in maintaining myofiber microtubule architecture and muscle function.

## Results

### The *Map4* transcript undergoes alternative splicing to yield a striated muscle-specific mMAP4

We analyzed human RNA-sequencing data from the NIH Epigenetic Roadmap Project (2008) and found that Map4 was the most abundant Map mRNA and protein in skeletal muscle (Figure 1B). To determine the proportion of Map4 transcripts that include *Map4* exon 8 in mouse tissues, we used multiplexed RT-PCR (Figures S1B and S1C). Exon 8 inclusion was detected predominantly in all striated muscle samples tested (heart, quadriceps, gastrocnemius) (Figure 1C) with percent spliced in (PSI) quantification ranging from 59% to 61% (Figure 1D), while uMAP4 was expressed across most tissues. Western blot analysis verified the expression of the mMAP4 protein (∼220 kDa) in striated muscle, which migrates slower than the expected 230 kDa (Figure 1E). The mMAP4 band accounted for 52–65% of the total MAP4 protein expressed in skeletal muscle. The minor bands are likely to be Map4 isoforms arising from minor levels of alternative splicing of additional exons.^22^^,^^23^^,^^24^ These data show that both uMap4 and mMap4 transcripts are expressed in skeletal muscle samples from mice and that the muscle-specific mMAP4 isoform accounts for over 50% of the total MAP4 protein. The abundance of the mMAP4 protein isoform and its expression in mature myofibers suggests that mMAP4 isoform plays an important role in the regulation of myofiber function.

### *Map4*^ΔE8/ΔE8^ mice show an increase in uMAP4, but no change in total MAP4 levels in skeletal muscle

**Figure 2:**
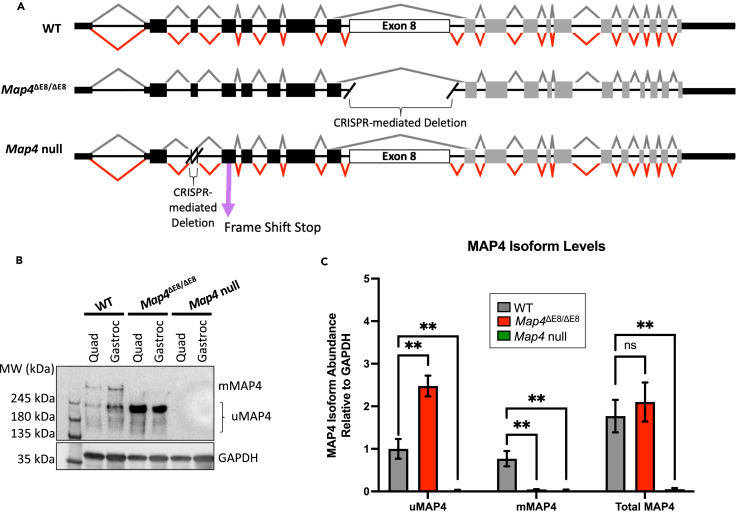
*Map4*^ΔE8/ΔE8^ mice show an increase in uMAP4, but no change in total MAP4 levels in skeletal muscle (A) Schematic representation of the Map4 genes in each of the CRISPR-generated mice. Double crosshatch indicates regions removed using CRISPR. Pink arrow on the Map4 null diagram indicates the location of the in-frame stop codon in exon 4 resulting from the CRISPR deletion in exon 3. (B) Representative western blot (1 lane per tissue for each genotype) using an antibody recognizing the various MAP4 isoforms (Anti-MAP4 SG-10) in skeletal muscle from each of the generated mice. (C) Quantitation of western blot analysis of gastrocnemius protein samples showing MAP4 protein levels in *Map4*^ΔE8/ΔE8^ and *Map4* null mice (statistical comparisons made by one-way ANOVA). *n* = 3 mice per genotype. Error Bars = SEM. [∗*p* < 0.05, ∗∗*p* < 0.01, ns = not significant].

To investigate the function of mMAP4 in skeletal muscle, we performed a CRISPR-mediated genomic deletion of Map4 exon 8 and generated mice that are homozygous for the deleted allele (*Map4*^ΔE8/ΔE8^) (Figure 2A). To compare the effects caused by loss of the mMAP4 isoform to the total loss of MAP4, we also generated CRISPR-mediated genomic deletions within exon 3 that cause a frameshift and a premature translation stop codon in exon 4 yielding *Map4* null mice when homozygous for the deleted allele (Figure 2A). Two independent founder lines for each of the *Map4*^ΔE8/ΔE8^ and *Map4* null alleles were generated. Given that founder animals may exhibit chimerism for additional mutations at the target site, each line was established from an individual F1 animal. In each case, the genomic fusion site was PCR amplified and sequenced to validate the removal of the targeted regions (Figure S2). RT-PCR analysis of skeletal muscle RNA from at least two homozygous animals of both genders confirmed correct splicing of exon 6 through exon 10 for *Map4*^ΔE8/ΔE8^ and 100% inclusion of the internally truncated exon 3 in Map4 null animals (data not shown). To mitigate the potential impact of off-target hits, each mouse line was backcrossed with wild type (WT) animals through at least six generations. Each of the two *Map4*^ΔE8/ΔE8^ and *Map4* null lines were shown to have comparable phenotypes and one line of each genotype was selected for analyses presented here (lines B878 and E584, respectively).

Western blot analysis of gastrocnemius and quadricep samples taken from all three mouse genotypes indicates loss of mMAP4 protein in skeletal muscle tissues of *Map4*^ΔE8/ΔE8^ mice and complete loss of MAP4 protein in the *Map4* null mice. *Map4*^ΔE8/ΔE8^ mice also displayed the expected concomitant increase of the uMAP4 isoform given the transition of mMAP mRNA to uMAP mRNA, but without a change in total MAP4 protein expression (Figures 2B and 2C).

### *Map4*^ΔE8/ΔE8^ mice have muscle weakness without evidence of histopathology

**Figure 3:**
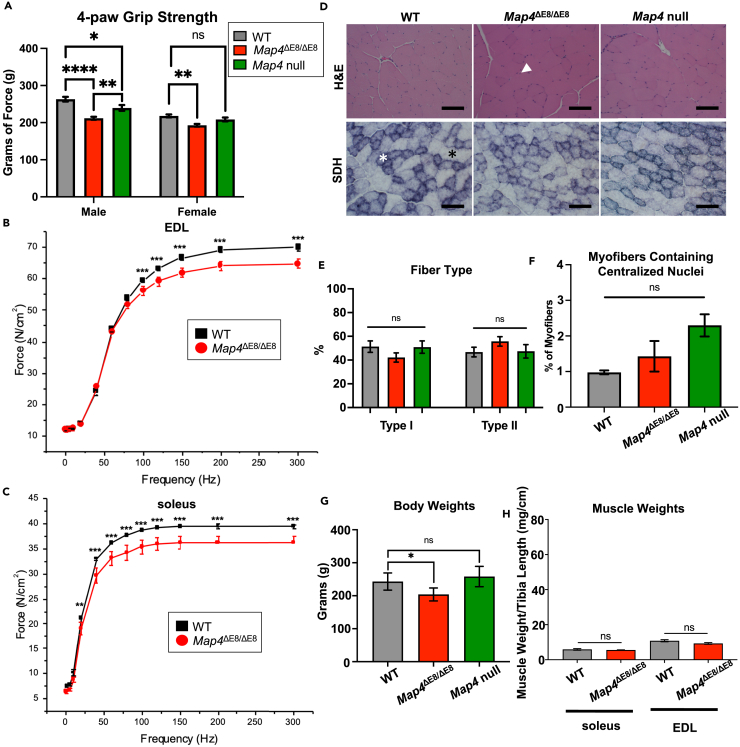
*Map4*^ΔE8/ΔE8^ mice have muscle weakness without evidence of histopathology (A) 4-paw grip strength of mice from each genotype at 10 months of age (statistical comparisons by one-way ANOVA). N = 7–12 mice per genotype. Error Bars = SEM. (B and C) *Ex vivo* force generation of the EDL and soleus of mice 10 months of age. *n* = 7 mice per genotype. Error Bars = SEM. (D) Representative histological staining of gastrocnemius cross-sections stained by hematoxylin and eosin (H and E) or succinate dehydrogenase (SDH). White arrowhead represents a centralized nucleus, the white star represents a type I fiber, and the black star represents a type II fiber. Scale Bar = 100 μm. (E–G) Quantification of gross features of the skeletal muscle in each genotype (statistical comparisons made by unpaired t-test). Error Bars = SEM. ∼300 cells from *n* = 3 mice for E-F, *n* = 3 mice for G. (H) Comparison of soleus and EDL muscle weights between WT and *Map4*^ΔE8/ΔE8^ mice. n = at least 7 mice per genotype. Error Bars = SEM. [∗*p* < 0.05, ∗∗*p* < 0.01, ∗∗∗*p* < 0.001, ∗∗∗∗*p* < 0.0001, ns, not significant].

To determine whether *Map4*^ΔE8/ΔE8^ and *Map4* null mice have a loss of muscle function, we tested 4-paw grip-strength in 10-week-old animals compared to corresponding WT mice controls derived from the same mating scheme as the experimental mice (Figure 3A). *Map4*^ΔE8/ΔE8^ mice showed a statistically significant decrease in grip strength in both males and females. *Map4* null mice had significantly reduced grip strength in males and while females showed a trend toward decreased grip strength, the results did not reach significance. Because grip strength was diminished more robustly in male mice, we chose to use males in subsequent experiments.

To precisely characterize the muscle weakness phenotype, we used *ex vivo* force analysis of individual muscles and directly compared 10-week-old *Map4*^ΔE8/ΔE8^ and WT mice. We analyzed force generation profiles of the extensor digitorum longus (EDL) and soleus muscles. We chose these two muscle groups because they represent the physiological extremes of skeletal muscle tissue metabolism. The EDL is composed largely of fast twitch, glycolytic myofibers while the soleus has the highest fraction of slow twitch, oxidative myofibers found in mice. We stimulated muscle contraction at different frequencies and measured the force of contraction. In both muscle groups, *Map4*^ΔE8/ΔE8^ muscles had significantly lower force generation that worsened as contraction frequency increased (Figures 3B and 3C). These results, coupled with the reduction in grip strength, show that *Map4*^ΔE8/ΔE8^ mice have impaired muscle contractile function.

To determine if the muscle weakness was associated with cell loss or muscle atrophy, we performed histology on the gastrocnemius muscle of *Map4*^ΔE8/ΔE8^, *Map4* null, and WT mice. We used hematoxylin and eosin (H&E) staining to assess myofiber structure and to identify myofibers with centralized nuclei as an indication of myopathy. We used succinate dehydrogenase (SDH) staining to assess shifts in oxidative capacity. Across both H&E and SDH staining we observed no difference in *Map4*^ΔE8/ΔE8^ or *Map4* null compared to WT controls (Figures 3D–3F). Despite the lack of muscle histopathology, *Map4*^ΔE8/ΔE8^ mice exhibited decreased body weight compared to WT mice (Figure 3G). To determine if this difference was due to loss of muscle mass, we compared the muscle weights of the muscles isolated in the *ex vivo* force generation assay between *Map4*^ΔE8/ΔE8^ and WT mice (Figures 3H and S3). We saw no significant differences in muscle weights. These results, along with the normal histology, demonstrates that *Map4*^ΔE8/ΔE8^ skeletal muscle weakness is not due to atrophy indicating an intrinsic molecular change within myofibers.

### Microtubules are disorganized in *Map4*^ΔE8/ΔE8^ skeletal muscle myofibers

To assess molecular changes in *Map4*^ΔE8/ΔE8^ mice, we examined the microtubule organization within myofibers isolated from the flexor digitorum brevis (FDB) following immunofluorescent staining. We stained using a mMAP4 specific antibody generated against the exon 8 protein region and co-stained microtubules using an alpha-tubulin antibody. Using 3 μm z-projected images taken at both the surface and core of the myofibers, we found that mMAP4 co-localizes with microtubules throughout the myofiber (Figure S4).

**Figure 4:**
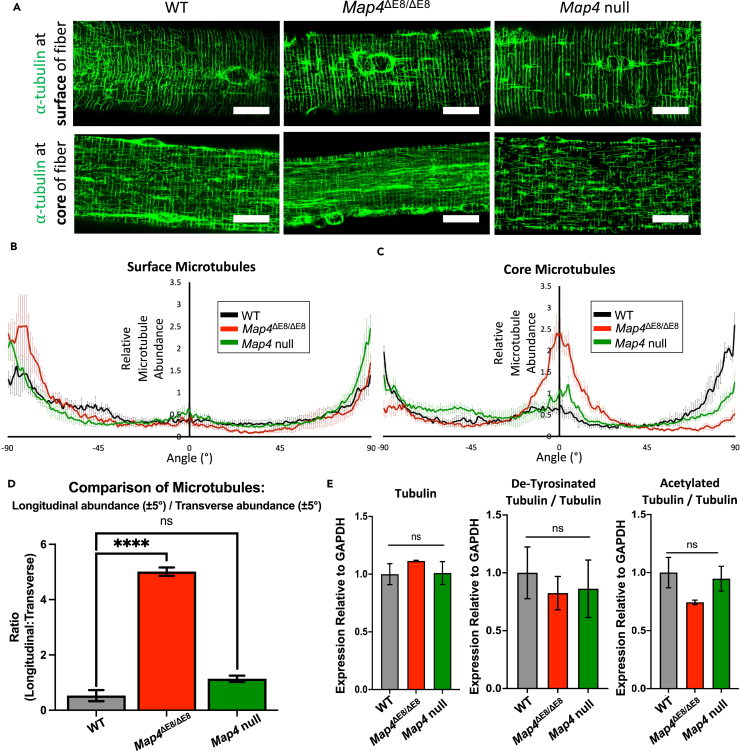
Microtubules are disorganized in *Map4*^ΔE8/ΔE8^ skeletal muscle myofibers (A) Representative alpha-tubulin staining showing a change in the microtubule orientation in *Map4*^ΔE8/ΔE8^ fibers. Surface microtubules visualized in z-projection of 2 μm from the surface. Core microtubules visualized in z-projections of 2 μm from the center. Scale Bar, 10 μm. Quantification of microtubule orientations at the (B) surface and (C) core of fibers. *n* = 5 cells/mice per genotype. Error Bars = SD. (D) Ratio of core microtubules oriented in the longitudinal and transverse directions (statistical comparisons made by an unpaired t-test). *n* = 5 cells/mice per genotype. Error Bars = SEM. (E) Quantitative western blot of beta-tubulin and two tubulin modifications associated with microtubule stability in gastric muscle samples. (statistical comparisons made by an unpaired t-test). *n* = 2 mice per genotype. Error Bars = SEM. [∗∗∗∗*p* < 0.0001, ns = not significant].

We noticed that microtubule organization was altered in *Map4*^ΔE8/ΔE8^ myofibers. We first imaged microtubules throughout myofibers and determined that microtubules at the surface differed visually than at the core (Video S1). To quantify microtubule organization, we used immunofluorescence microscopy and a combination of image analysis methods to plot relative microtubule orientation at the surface and the core of the myofiber.^25^^,^^26^ Because myofiber microtubules are largely transverse or longitudinal relative to the myofiber axis, longitudinal microtubules were set at 0° and transverse microtubules were set at ± 90° (Figure S5A). Surface microtubules showed no significant differences in myofibers isolated from *Map4*^ΔE8/ΔE8^, *Map4* null, and WT mice (Figures 4A and 4B). However, we found a significant increase in the ratio of longitudinal to transverse of the core microtubules between WT and *Map4*^ΔE8/ΔE8^ core myofiber microtubules (Figures 4A–4D). These results indicate that microtubules are selectively disorganized in *Map4*^ΔE8/ΔE8^ myofibers.

Video S1. Representative Video of myofibers isolated from the FDB of WT and Map4ΔE8/ΔE8 mice The video displays alpha-tubulin staining slice-by-slice from the surface through to the core of the myofibers. Each slice is 1 μm thick. Bar, 10 μm.

Microtubules can become disorganized when tubulin levels change or when microtubule stability is affected by tubulin posttranslational modifications. To determine whether changes in Map4 expression affected tubulin or tubulin posttranslational modifications, we measured beta-tubulin protein levels in skeletal muscle in each of the genotypes. We included the analysis of de-tyrosinated tubulin and acetylated tubulin as markers of increased microtubule stability.^27^^,^^28^ There was no significant change in tubulin protein levels and no changes in de-tyrosinated or acetylated tubulin across the three genotypes, suggesting that *Map4*^ΔE8/ΔE8^ myofiber microtubules are disorganized by a mechanism independent of tubulin stability (Figures 4E and S5B).

### Increased expression of uMAP4 induces microtubule disorganization in myofibers

As mentioned previously*, Map4*^ΔE8/ΔE8^ myofibers do not express increased total MAP4 compared to WT but have higher levels of uMAP4 since all *Map4* mRNAs lack exon 8 (Figure 2C). To understand the importance of uMAP4 and mMAP4 differential splicing, we tested whether muscle weakness and disrupted microtubule architecture result from the loss of mMAP4, the gain of additional uMAP4, or a combination of both.

**Figure 5:**
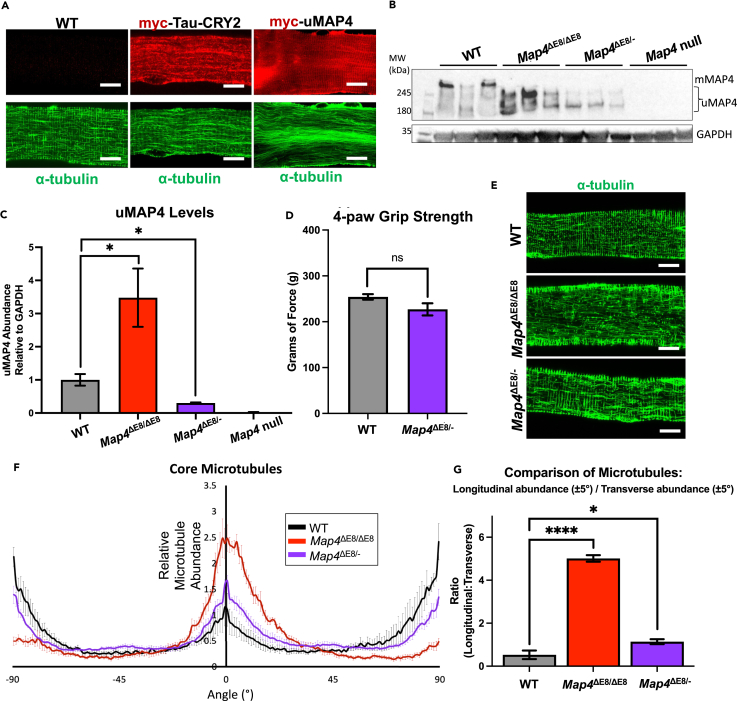
Increased expression of uMAP4 induces microtubule disorganization in myofibers (A) Representative immunofluorescent staining of myofiber cores with similar average intensities of Myc (red) and alpha-tubulin (green) comparing wild-type myofibers to myofibers over-expressing myc-uMAP4 or a myc-Tau-CRY2 control. Myofibers were isolated six days following plasmid electroporation. Scale Bar, 10 μm. (B) Western blot (3 replicates per genotype) using an antibody recognizing all MAP4 isoforms (Anti-MAP4 SG-10) in gastrocnemius skeletal muscles from the indicated mouse genotypes. (C) Quantification of the western blot. *n* = 3 mice per genotype. (D) Grip strength analysis on WT and *Map4*^ΔE8/-^ mice. n = at least 7 mice per genotype. (E) Representative immunofluorescent staining of alpha-tubulin comparing WT mouse myofiber cores to *Map4*^ΔE8/ΔE8^ and *Map4*^ΔE8/-^ mice. Scale Bar, 10 μm. (F) Quantification of microtubule orientations. n = at least 4 cells/mice per genotype. Error Bars = SD. (G) Ratio of longitudinal and transverse microtubules in myofiber cores. n = at least 4 per genotype. Error Bars = SEM.

Two methods were used to determine whether increased uMAP4 levels had a detrimental effect in myofibers. First, we used plasmid electroporation to over-express myc-tagged uMAP4 in WT mouse FDB muscle and co-stained isolated myofibers for myc-uMAP4 and alpha-tubulin to look for signs of microtubule disorganization. Previous studies have used electroporation to express proteins from plasmids in the myofibers of the FDB.^29^^,^^30^ However, we found that plasmid electroporation of the FDB induced a basal level of damage to microtubule organization in myofibers proportional to the plasmid size (data not shown). Therefore, a related MAP of similar size, myc-tagged Tau-CRY2, was used as a control for potential cell damage resulting from the overexpression of any MAP or from the electroporation. To control for differences in expression, myofibers with comparable average fluorescent intensities using anti-myc antibody were used for analysis. We found that exogenous expression of uMAP4 disrupted myofiber microtubule organization when compared to myofibers over-expressing myc-Tau-CRY2 (Figure 5A). These results strongly suggested that increased uMAP4 levels disrupts myofiber microtubule organization.

We hypothesized that if increased uMAP4 levels in *Map4*^ΔE8/ΔE8^ mice contributed to the phenotype, reduced expression of uMAP4 in the *Map4*^ΔE8/ΔE8^ background would rescue muscle weakness and microtubule disruption. To reduce uMAP4 expression in *Map4*^ΔE8/ΔE8^ mice, we crossed *Map4*^ΔE8/ΔE8^ and *Map4* null mice to generate *Map4*^ΔE8/-^ mice that have one copy each of the exon 8 deletion and the null allele. These mice lack mMAP4 protein and contain only one copy of the uMAP4 allele. Western blot analysis showed a significant decrease in uMAP4 levels in *Map4*^ΔE8/-^ mice compared to *Map4*^ΔE8/ΔE8^ mice (Figures 5B and 5C). 4-paw grip strength analysis of 10-week-old *Map4*^ΔE8/-^ mice indicated no difference in muscle strength compared to WT mice (Figure 5D) indicative of a rescue of the muscle weakness observed in *Map4*^ΔE8/ΔE8^. To determine if microtubule architecture was rescued in *Map4*^ΔE8/-^ FDB myofibers we quantified the longitudinal and transverse microtubule orientation. Quantitative analysis of *Map4*^ΔE8/-^ and *Map4*^ΔE8/ΔE8^ myofiber microtubules showed a partial rescue of microtubule organization compared to WT (Figures 5E–5G). These results support the hypothesis that increased uMAP4 levels has deleterious effects on muscle strength and on myofiber microtubule organization. Given that total levels of MAP4 are not affected in *Map4*^ΔE8/ΔE8^ mice (Figure 2C), it is the specific increase of the uMAP4 isoform that contributes to muscle weakness and disorganized microtubules.

### Exon 8 promotes tighter co-localization of MAP4 proteins with microtubules

**Figure 6:**
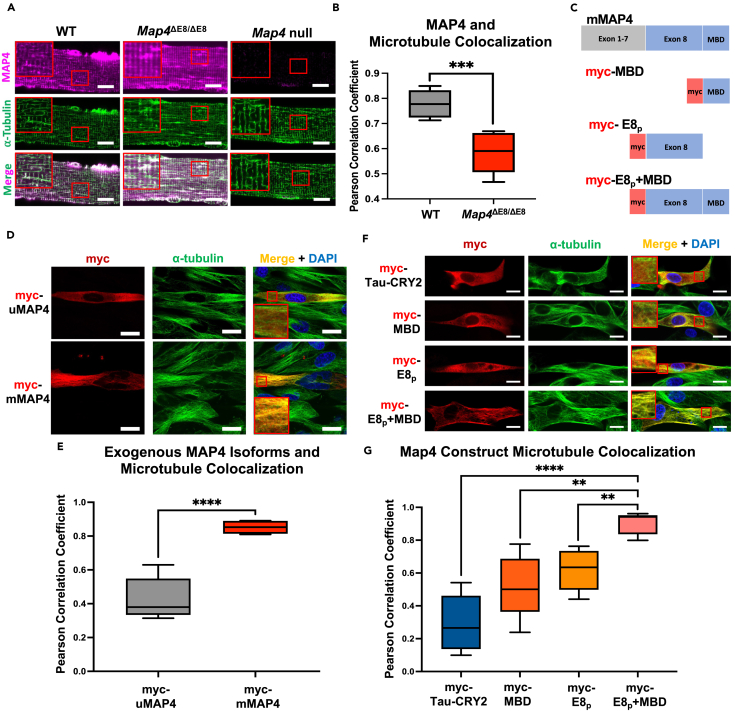
Exon 8 promotes tighter co-localization of MAP4 proteins with microtubules (A) Representative immunofluorescent staining of total MAP4 co-stained for alpha-tubulin. Enlarged insets are shown in red box. Bar, 10 μm. (B) Average Pearson correlation coefficient to quantify colocalization of the endogenous MAP4 and microtubule signals. *n* = 3 myofibers/mice per genotype. Error Bars = min and max values. (C) Schematic representation of the myc-tagged mMAP4 constructs used in transfections. MDB = microtubule binding domain. (D) Representative immunofluorescent staining of exogenous myc-MAP4 isoforms (red) co-stained for alpha-tubulin (green). Enlarged insets are shown in red box. Bar, 10 μm. (E) The average Pearson correlation coefficient for colocalization of the exogenous myc-MAP4 isoforms with microtubules. *n* = 4. Error Bars = min and max values. (F) Representative immunofluorescent staining of myc-tagged constructs (red) co-stained for alpha-tubulin (green). Enlarged insets are shown in red box. (G) The average Pearson correlation coefficient for colocalization of various myc-tagged constructs with microtubules. *n* = 4. Error Bars = min and max values. For all Pearson correlation coefficients, 1 indicates perfect correlation. All statistical comparisons made by an unpaired t-test. [∗∗*p* < 0.01, ∗∗∗*p* < 0.001, ∗∗∗∗*p* < 0.0001].

The results above indicated that the gain of uMAP4 has a detrimental effect in skeletal muscle; we next sought to characterize the role of mMAP4 in microtubule regulation and determine whether loss of the mMAP4 isoform also contributed to the *Map4*^ΔE8/ΔE8^ phenotypes. To investigate potential differences between mMAP4 and uMAP4 *in vivo*, we first compared the degree to which uMAP4 and mMAP4 co-localize with microtubules. We performed immunofluorescent staining of MAP4 and compared staining patterns between WT and *Map4*^ΔE8/ΔE8^ mice (Figure 6A). MAP4 staining in WT myofibers was moderately co-localized with tubulin staining, as quantified by the Pearson correlation coefficient (Figures 6B, S6A, and S6B). However, the Pearson correlation coefficient for MAP4 co-localization with microtubules in *Map4*^ΔE8/ΔE8^ myofibers was significantly lower than WT. These results indicate that despite the increased level of uMAP4 in *Map4*^ΔE8/ΔE8^ myofibers there is significantly less association of endogenous uMAP4 with microtubules compared to endogenous mMAP4 and uMAP4 in WT myofibers (Figures 6B, S6A, and S6B). This result is consistent with the relatively poor co-localization of exogenously expressed uMAP4 with microtubules *in vivo* which is apparent in Figure 5A.

We hypothesized that mMAP4 exhibits higher affinity to microtubules compared to uMAP4. To investigate the impact of the exon 8-encoded protein on association with microtubules in a cellular context, we overexpressed different myc-tagged derivatives of MAP proteins in an immortalized human myoblast line (LHCN).^31^ The proteins tested included uMAP4, mMAP4, the protein region encoded by exon 8 (E8_p_) and E8_p_ fused to the downstream microtubule-binding domain (E8_p_+MBD) (Figure 6C). We found that myc-uMAP4 had poor co-localization with endogenous microtubules while mMAP4 showed substantial co-localization (Figures 6D and 6E). Next, we sought to determine which region of mMAP4 is required to promote the higher degree of co-localization to microtubules and tested the association of myc-tagged MBD, E8_p_ and E8_p_+MBD with microtubules in LHCN cells (Figure 6F). Each construct, along with the myc-Tau-CRY2 control construct, was transfected into LHCN cells and stained for myc and alpha-tubulin to visualize co-localization between the myc-tagged protein and microtubules. Of the four constructs tested, myc-E8+MBD displayed the strongest level co-localization (Figures 6F and 6G), nearly as strong as the full-length mMAP4 (Figure 6E). These results indicate that mMAP4 has a high propensity for microtubule co-localization when E8_p_ is connected directly to the MBD.

To directly compare the binding of MAP4 isoforms expressed in WT vs. *Map4*^ΔE8/ΔE8^ mouse skeletal muscle. Using magnetic beads conjugated to a MAP4 antibody (G-10) that recognizes common epitopes, we performed immunoprecipitation pull-downs of MAP4 in each tissue. We then mixed the beads with bound MAP4 protein with pure Rhodamine-labeled tubulin (Rh-Tub) and allowed the samples to incubate at 37°C for 20 min. The beads were washed to remove tubulin that did not bind to MAP4. Bead-bound MAP4 Rh-Tub was boiled to release proteins that were run on SDS-PAGE (Figure S7A). The amount of Rh-labeled tubulin bound to the MAP4 derived from either WT or *Map4*^ΔE8/ΔE8^ mice was then quantified by Rhodamine fluorescence. Because more total MAP4 bound to the beads in the *Map4*^ΔE8/ΔE8^ samples (Figure S7B), tubulin levels in each sample were normalized to the total amount of MAP4 pulled down. We found that in WT mice, where both uMAP4 and mMAP4 were bead-bound and mMAP4 predominates, tubulin binding was more than two times higher than in the *Map4*^ΔE8/ΔE8^ samples containing only uMAP4 (Figure S7C). These results support that the presence of mMAP4 increases tubulin interaction compared to when uMAP4 is alone.

## Discussion

In this study, we highlighted the roles of uMAP4 and mMAP4 in maintenance of microtubule organization and myofiber function. We show that *Map4*^ΔE8/ΔE8^ mice display significant skeletal muscle weakness and increased longitudinal and decreased transverse microtubule density in myofibers. Notably, deletion of exon 8, thereby removal of the mMAP4 isoform, resulted in concomitant elevation of uMAP4 levels, and a subsequent detrimental effect on microtubule organization and skeletal muscle force generation. While the exact mechanisms for this negative effect are unknown, previous studies have linked uMAP4 to the regulation of microtubule dynamics and its interaction with cargo and motor proteins.^18^^,^^19^^,^^20^ Therefore, it is possible that elevated levels of uMAP4 could impede cellular transport processes leading to poor myofiber health. In addition, our findings underscore the significance of alternative splicing in regulating protein isoform ratios that are appropriate for tissue function. Specifically, alternative splicing of exon 8 may serve as a biological buffer in myofibers, modulating uMAP4 abundance to balance its function against potential dysfunction. Analogous regulatory mechanisms are observed in the brain with the *Mapt* gene (Tau), that shares a similar gene structure to MAP4 and undergoes multiple alternative splicing events that, when the ratios are altered, are associated with neurodegeneration.^32^^,^^33^^,^^34^^,^^35^

We demonstrated that mMAP4 exhibits superior co-localization with microtubules compared to uMAP4. Interestingly, neither the MBD nor E8_p_ alone showed significant co-localization with microtubules, but the combination of E8_p_+MBD displayed robust co-localization. Structure prediction software such as AlphaFold predict E8_p_ and the MBD to be intrinsically disordered. Intrinsically disordered proteins are amenable to rapid interactions that induce different protein structures.^36^^,^^37^ E8_p_ may interact with the MBD to induce a folding allowance that has a higher association with microtubules. Of interest for future studies are how E8_p_ interacts with the MBD and whether this interaction stabilizes the MBD while bound to microtubules or if it promotes a conformation that has a higher affinity to microtubules.

Overall, our study highlights the critical role of mMAP4 in myofibers, acting as a buffer against elevated uMAP4 levels and exhibiting tighter association with microtubule networks. The differential microtubule interactions of the two MAP4 isoforms offer insights into microtubule regulation. Given the dynamic nature of microtubules, it is essential that different MAPs can bind and dissociate from microtubules at various rates to promote different levels of stability. This would imply that the ratio of uMAP4 and mMAP4 isoforms may assist in the modulation of microtubule dynamics. Alternative splicing of the Map4 transcript can provides a biological system that allows the cell to control microtubule regulation and organization by modulating microtubule dynamics when needed. This regulatory mechanism is particularly relevant in the postnatal transition of increased mMAP4 expression and in myoblast differentiation, where shifts in MAP4 isoform production may influence cytoskeletal reorganization.^17^

### Limitations of the study

Our use of *in vivo* models identified the physiological significance of the muscle-specific mMap4 isoform for skeletal muscle force generation and microtubule architecture. However, this study did not investigate the basis for the increased association of mMAP4 with microtubules compared to uMAP4. Additional studies into the structural insights of mMAP4-microtubule interactions would require *in vitro* analyses.

## Resource availability

### Lead contact

Further information and requests for resources and reagents should be directed to and will be fulfilled by the lead contact, T.A.C. (tcooper@bcm.edu).

### Materials availability

Mouse lines and plasmids generated in this study and are maintained and frozen on-site at Baylor College of Medicine. Any additional information or requests to access these materials should be made to the lead contact.

### Data and code availability

• Data: All data reported in this paper will be shared by the lead contact upon request.
• Code: This paper does not report any original code.
• All other items: Any additional information required to reanalyze the data reported will be shared by the lead contact upon request.

## Acknowledgments

This project was supported by funding from 10.13039/100000002NIH R01HL147020, 10.13039/100000002NIH R01AR060733 and R01AR082852 (TAC). This project was supported by the Optical Imaging & Vital Microscopy Core at the Baylor College of Medicine. We thank the Genetically Engineered Rodent Models (GERM) Core at Baylor College of Medicine for work in generating the Map4 ΔE8/ΔE8 and Map4 null mice. The GERM Core is funded in part by the National Institutes of Health Cancer Center Grant (P30 CA125123).

For general data regarding MAP expression in muscle, we thank the many scientists who have contributed data to the Epigenetics Roadmap Project (https://nihroadmap.nih.gov/epigenomics/). We also thank the Optical Imaging & Vital Microscopy Core at Baylor College of Medicine for advanced training and techniques in microscopy. Additionally, we thank Josh Sharp for initiating the project, and Dr. Josephine Ferreon, and Dr. Phoebe Tsoi for their expertise and experimental support throughout the development of the project. Lastly, we thank Sara Johnson for her assistance in editing and members of the Thomas Cooper lab for their assistance either physically, intellectually, or emotionally throughout the study.

## Author contributions

Conceptualization, L.L and T.A.C.; methodology L.L., L.N., and T.A.C., formal analysis L.L., B.N., J.A.L., and G.G.R.; investigation, L.L., L.N., B.N., and G.G.R.; writing – original draft, L.L.; writing – review and editing, L.L., L.N., and T.A.C.; visualization, L.L. and T.A.C.; supervision, T.A.C.; project administration, T.A.C.; funding acquisition, T.A.C.

## Declaration of interests

The authors declare that they have no competing interests.

## STAR★Methods

### Key resources table

**Table:** 

REAGENT or RESOURCE	SOURCE	IDENTIFIER
**Antibodies**		

Mouse anti-MAP4 (G-10)	Santa Cruz	catalog number sc-390286; RRID: AB_3662746
Mouse anti-Beta-Tubulin (D-10)	Sanat Cruz	catalog number sc-5274; RRID: AB_2288090
Mouse anti-Detyrosinated Alpha-Tubulin	AbCam	catalog number ab254154; RRID: AB_3662747
Mouse anti-Acetylated Alpha-Tubulin	Santa Cruz	catalog number sc-23950; RRID: AB_628409
Rabbit anti-GAPDH	Cell Signaling	catalog number 14C10; RRID: AB_561053
Rabbit anti-mMAP4	This Paper	RRID: AB_3662748
Anti-myc-Alexa fluorophore 555	Milipore Corp.	Catalog number 16-225; RRID: AB_442400
Anti-alpha-tubulin-Alexa fluorophore 488	Invitrogen	Catalog number 322588; RRID: AB_2532182

**Bacterial and virus strains**		

Stellar™ Competent Cells	Takara	Catalog number 636763

**Critical commercial assays**		

Lipofectamine™ 3000 Transfection Reagent	ThermoFisher	Catalog number: L3000001
In-Fusion® Snap Assembly Master Mix	Takara	Catalog number: 638948
Rhodamine-labeled tubulin	Cytoskeleton Inc	Catalog number: BST01-001
100 mM GTP	Cytoskeleton Inc	Catalog number: BST06-001
1X General tubulin buffer	Cytoskeleton Inc	Catalog number: BST01-001

**Experimental models: Cell lines**		

Pectoralis myogenic clone no. 2 (LHCN-M2)	Zhu et al.^31^	N/A

**Experimental models: Organisms/strains**		

Friend leukemia virus B (FVB)	The Jackson Lab	Strain #:001800; RRID:IMSR_JAX:001800
*Map4*^ΔE8/ΔE8^	This Paper	N/A
*Map4*^ΔE8/-^	This Paper	N/A

**Oligonucleotides**		

CRISPR MAP4 Exon 8 sgRNA A 5′-AAACACCTCTTATCCTTAACTGG-3′	This Paper	N/A
CRISPR MAP4 Exon 8 sgRNA B 5′- GCCAACAGACTAGCGATCAATGG-3′	This Paper	N/A
CRISPR MAP4 Exon 8 sgRNA C 5′-ATTGTTGATAAACCCAGTTAAGG-3′	This Paper	N/A
CRISPR MAP4 Exon 8 sgRNA D 5′-TCCATTGATCGCTAGTCTGT TGG-3′	This Paper	N/A
CRISPR MAP4 Exon 3 sgRNA A 5′-CTCCTAGCCAATGGTGATCATGG-3′	This Paper	N/A
CRISPR MAP4 Exon 3 sgRNA B 5′-TTCCATGATCACCATTGGCTAGG-3′	This Paper	N/A
RT-PCR Primers Map4 exon 6-7 junction 5′-GGCTTCACCAGAACCAGTCA-3′	This Paper	N/A
RT-PCR Primers Map4 exon 8 5′-CCAGTAGCTCAGGACCCAC-3′	This Paper	N/A
RT-PCR Primers Map4 exon 10-11 junction 5′-TTGGCTTTGGCTTCACGTCTC-3′	This Paper	N/A

**Recombinant DNA**		

pcDNA™3.1 (+) Mammalian Expression Vector	Invitrogen	Catalog number V79020
Tau-CRY2	Gift	N/A
Cloned MAP4 Constructs into pcDNA™3.1 (+) Mammalian Expression Vector	This Paper	N/A

**Software and algorithms**		

Fiji-2	https://imagej.net/software/fiji/downloads	N/A
OrientationJ	https://bigwww.epfl.ch/demo/orientation/	N/A
Image Studio Lite	https://www.licor.com/bio/image-studio-lite/	N/A

### Experimental model and study participant details

#### Mouse husbandry

The mice in this study were cared for in the animal facilities located at Baylor College of Medicine, adhering to a 12-h light/dark cycle, and were housed in groups ranging from 1 to 5 mice. All mice received weekly cage changes and were monitored daily for any signs of distress or poor health. All animal care and experimental protocols received approval from the Institutional Animal Care and Use Committee (IACUC) of Baylor College of Medicine, in accordance with the guidelines of the US National Institutes of Health.

#### Generation of mice

*Map4*^ΔE8/ΔE8^ mice were generated in an inbred albino Friend leukemia virus B (FVB) background through collaboration with the BCM Genetically Engineered Rodent Model (GERM) Core. The elimination of alternatively spliced Map4 exon 8 was achieved using four single-guide RNAs (sgRNAs) selected by the mES Core. These sgRNAs were identified through the Wellcome Trust Sanger Institute Genome Editing website (http://www.sanger.ac.uk/htgt/wge/), positioned to flank the genomic region containing Map4 exon 8. The sgRNAs (A-D) are as follows: A 5′-AAACACCTCTTATCCTTAACTGG-3’ (TGG is PAM sequence); B 5′- GCCAACAGACTAGCGATCAATGG-3’ (TGG is PAM sequence); C 5′-ATTGTTGATAAACCCAGTTAAGG-3’ (AGG is PAM sequence); D 5′-TCCATTGATCGCTAGTCTGT TGG-3’ (TGG is PAM sequence).^38^

Map4 null mice were also generated within an FVB background using identical methods, however deletion within Map4 exon 3 was achieved using two sgRNAs. The sgRNAs (A-B) are as follows: A 5′-CTCCTAGCCAATGGTGATCATGG-3’ (TGG is the PAM sequence); B 5′-TTCCATGATCACCATTGGCTAGG-3’ (AGG is the PAM sequence).

Two independent founder lines were generated for both *Map4*^ΔE8/ΔE8^ (B863 and B878) and *Map4* null (E584 and E587) mice subsequent to screening progeny obtained from 2 to 5 founder animals. Each of the four lines were ultimately generated from an F1 animal, the genomic deletions of which were confirmed by sequencing of PCR products (Figure S2 and data not shown). Mice underwent 6 back crosses before being used for experiments.

Male *Map4*^ΔE8/ΔE8^ mice displayed a more robust grip-strength phenotype compared to females (Figure 3A). We therefore used male mice for the data collection throughout this experiment. Early on, we tested grip strength of WT and *Map4*^ΔE8/ΔE8^ mice at different ages (8 weeks–10 months) and saw robust muscle weakness in *Map4*^ΔE8/ΔE8^ mice at every age tested (data not shown). To maintain consistency in experiments, we chose to use mice approximately 10 months of age for all experiments in this study.

#### Growth and maintenance of cells

For experiments involving cell culturing techniques, we used immortalized human skeletal myoblasts derived from the pectoralis myogenic clone no. 2 (LHCN-M2).^31^ These are myogenic stem cells (satalite cells) that were isolated from the pectoralis muscle of a 41-year-old male patient. The cell lines are routinely validated using muscle-specific markers in the lab and are subject to differentiation regularly to ensure they maintain their skeletal-muscle-like qualities.

LHCN-M2 Cells were maintained in growth medium containing DMEM (ThermoFisher Scientific) supplemented with 15% FBS (GeneDepot), 0.02M HEPES (Gibco), 0.03 μg/mL Zinc Sulfate (Sigma), 1.4 μg/mL Vitamin B12 (Sigma), 0.055 μg/mL Dexamethasone (Sigma), 2.5 ng/mL Hepatocyte Growth Factor, recombinant human (catalog number: GF116, EMD Millipore), and 10 ng/mL basic FGF-2, human recombinant (catalog number: 1140-02-500, Goldbio). Cells were grown and maintained at 37°C in 5% CO2.

### Method details

#### Analysis of NIH roadmap epigenetics project

All data collected and used in accordance with the required 9-month embargo period outlined by the NIH. The relative abundance of various MAP4 proteins was determined using “skeletal muscle upper limb” for skeletal muscle RNA and protein, “heart left ventricle” for cardiac muscle RNA, and “stomach” for smooth muscle RNA. All data was open access and is available here: https://www.ncbi.nlm.nih.gov/geo/roadmap/epigenomics/

#### Protein isolation from mouse tissues

All tissues were isolated from mice sacrificed in accordance with IACUC regulations. For skeletal muscle isolation, each muscle was isolated using ROBOZ RS-5910 dissection scissors and FDT 11254-20 fine tweezers. Upon removal of the skin from each leg, the tibialis anterior (TA) and extensor digitorum longus (EDL) were removed cutting at the tendon near the ankle and at the patella. The EDL was then separated from the TA. The quadriceps (quad) were removed by first removing the encasing fat followed by cutting along the femoral bone to release the quad. Lastly, the gastrocnemius (gastroc) and soleus were removed from the back of the leg by isolating the two muscle together and cutting along the tendon near the heel and the tendon located behind the patella. The soleus was then separated from the gastroc. All non-skeletal muscle tissues were collected from their corresponding locations and removed carefully as full tissues. Tissues were minced into homogeneous mixtures. Tissue homogenates were prepared from mice ranging from 9 to 11 weeks of age. Homogenization of each tissue was done in 1× RIPA buffer (Catalog number: 89900, Thermo Fisher Scientific) supplemented with 1× protease inhibitor (GenDepot) and 1× phosphatase inhibitor (GenDepot). The homogenization process involved combining the tissues with RIPA buffer and 1.0 mm diameter Zirconium Oxide Beads (Next Advance), followed by blending using the Bullet Blender 2.4 Tissue Homogenizer (Next Advance) for 3–9 min depending on the tissue with regular cooling on ice. Samples were incubated for 15 min at 4°C and then centrifuged at 15,000 × g for 10 min at 4°C. Protein concentrations in the supernatant were determined using the Pierce BCA Protein Assay Kit (Thermo Fisher Scientific).

#### Western blotting of tissue lysates

Tissue lysates were diluted and prepared in 4× Laemmli Sample Buffer (Bio-Rad) supplemented with 10% 2-Mercaptoethanol so that the final protein concentration was 3 μg/μL. Samples were heated to 95°C for 10-min 40 μg of protein was run on a 4–20% Criterion TGX Stain-Free Precast Protein Gel (Bio-Rad Laboratories) using Tris/Glycine/SDS Running Buffer (Bio-Rad Laboratories). The proteins were then transferred to Trans-Blot Turbo Nitrocellulose (Bio-Rad Laboratories) through the Trans-Blot Turbo system (Bio-Rad Laboratories) at 2.5 A, 25 V, 9 min in Trans-Blot Turbo Transfer Buffer (Bio-Rad Laboratories). After blocking for 1 h at room temperature with 5% Non-Fat Dry-Milk (Chem Cruz) in phosphate-buffered saline (10 mM Na_2_HPO_4_, 2.7 mM KCl, 137 mM NaCl, 1.76 mM KH_2_PO_4_, pH 7.4) with 0.01% Tween 20 (Sigma-Aldrich) (PBST), membranes were probed overnight at 4°C with their respective antibodies (anti-MAP4 (G-10) catalog number sc-390286, Santa Cruz, [1:250]; anti-Beta-Tubulin (D-10), catalog number sc-5274 Santa Cruz, [1:1000]; anti-Detyrosinated Alpha-Tubulin, catalog number ab254154, abcam, [1:1000]; anti-Acetylated Alpha-Tubulin, catalog number sc-23950, Santa Cruz [1:1000], or anti-GAPDH, catalog number 14C10, Cell Signaling, [1:10000]).

The following day, the blots were washed three times with PBST and incubated for 1 h at room temperature in the secondary antibody. The secondary antibody used to detect all mouse-generated primary antibodies was Horseradish Peroxidase (HRP)-conjugated Mouse IgG (H&L) Antibody (Jackson Immunoresearch, [1:10,000]) in 5% non-fat milk in PBST. The secondary antibody used to detect all rabbit-generated primary antibodies was HRP-conjugated Rabbit IgG (H&L) Antibody (Jackson Immunoresearch, [1:10,000]) in 5% non-fat milk in PBST. The membranes were then washed three times with PBST, incubated for 10–30 s in SuperSignalTM West Femto Maximum Substrate (Thermo Fisher Scientific), and finally imaged using the ChemiDox Xrs Molecular Imaging System (Bio-Rad). Quantification of bands in western blots was done using ImageStudio lite.

#### RNA isolation from mouse tissues

Total RNA was extracted from 9 to 11-week-old mouse tissues utilizing the RNeasy Fibrous Tissue Mini Kit (catalog number 74704; Qiagen) following the manufacturer’s guidelines. Tissue was homogenized using the Bullet Blender 2.4 Tissue Homogenizer (Next Advance) with 0.1 g of 0.5 mm diameter Zirconium Oxide Beads (Next Advance). Random-primed cDNA was synthesized from 1 μg of total RNA using the High Capacity cDNA Reverse Transcription Kit (catalog number 2751911; Invitrogen) according to the manufacturer’s instructions.

#### RT-PCR of total RNA isolated from tissues

Forward and reverse primers (either a single pair or multiplexed) were added to a final concentration of 1 μM to the cDNA derived from the RNA isolation. Samples were added to the amfiSure PCR Master Mix (catalog number P0311; GenDepot). After the PCR reaction was complete, samples were run on a 5% polyacrylamide gel (0.1 M Tris, 0.1 M Boric Acid, 2 mM EDTA, 5% acrylamide/bis 19:1 (catalog number 0496, VWR), 0.1% APS (catalog number 1610700, Bio-Rad), TEMED (catalog number 161–0801, Bio-Rad) in TBE running buffer (0.1 M Tris, 0.1 M Boric Acid, 2 mM EDTA). Gels were placed in a 0.8 μg/mL Ethidium Bromide solution for 10 min and imaged using a Gel Logic 2200 Imaging System (Kodak). When possible, primers were designed to bind exon junctions to ensure no genomic DNA was amplified during PCR. The forward primer sequence (binding the Map4 exon 6-7 junction) is 5′-GGCTTCACCAGAACCAGTCA-3’. The reverse primer sequence (binding within Map4 exon 8) is 5′-CCAGTAGCTCAGGACCCAC-3’. The reverse primer sequence (binding the Map4 exon 10-11 junction) is 5′-TTGGCTTTGGCTTCACGTCTC-3’.

#### Ex vivo force generation assay

Male mice were used for the dissection of soleus and EDL muscles. Employing 4-0 silk suture, one tendon of each muscle was securely tied to a fixed hook, while the other tendon was fastened to a force transducer (F30; Harvard Apparatus). Muscles were placed in a physiological saline solution containing (mM): 2.0 CaCl_2_, 120.0 NaCl, 4.0 KCl, 1.0 MgSO_4_, 25.0 NaHCO_3_,1.0 KH_2_PO_4_ and 10.0 glucose, pH 7.3, and continuously gassed with 95% O_2_–5% CO_2_ at 25°C. Muscles were then incubated at 30°C for 15 min and optimal muscle length (Lo) and voltage (Vmax) were adjusted to elicit maximal twitch force. To assess force-frequency, muscles were stimulated at frequencies of 1, 5, 10, 20, 40, 60, 80, 120, 150, and 200 Hz, with pulse and train durations of 0.5 and 250 ms at 1-min intervals. Muscle weight and Lo were used to estimate cross-sectional area, and absolute forces were expressed in newtons per centimeter squared.^39^

#### Grip strength assay and mouse body weight

4-paw grip strength assessment was performed on mice aged 9–11 weeks using a Columbus Instruments grip strength meter. Mice were allowed a minimum of 10 min to acclimate to the testing room to mitigate stress effects on behavior during the assessment. The experimenter was blinded to the mouse genotypes, and the same experimenter conducted all grip strength evaluations to minimize variability. Utilizing a digital force transducer, the peak pull force in grams was recorded as the mouse grasped the grid. This procedure was repeated for three trials and the results were averaged. After the third trial, animals were weighed.

#### Skeletal muscle weights

The weights of the tibialis anterior and gastrocnemius muscles from mice ranging from 9 to 11 weeks of age, were measured using an analytical balance and subsequently normalized to the tibia length. Each muscle was carefully dissected under a dissection scope to ensure the complete removal of fat and tendons.

#### Dissection of FDB and isolation of myofibers

Mice were anesthetized with 2% isoflurane and subsequently euthanized via cervical dislocation. The flexor digitorum brevis (FDB) tissue was surgically dissected by first removing connective tissue under the footpad and carefully cutting the FDB at the tendon near heel and near the digits. The FDB muscle was placed in wells of a 24-well plate containing minimum essential medium (catalog number 51411C, Sigma-Aldrich), 4 mg/mL collagenase type 1 (catalog number C0130, Sigma-Aldrich), and 0.1% gentamycin. It was then incubated in 5% CO2 at 37°C for 1 h. After incubation, the FDB muscle was transferred to a new well and individual fibers were gently released by serial trituration into 1 mL of DMEM (catalog number 11-965-092, Thermo Fisher Scientific) supplemented with 10% FBS (catalog number F0900-050, GenDEPOT) and 1% penicillin/streptomycin. The isolated myofibers were then placed into a 1.5 mL tube and allowed to settle before being washed in DPBS (catalog number CA008, GenDEPOT) and fixed in 10% formalin (catalog number HT501128, Sigma-Aldrich) and subsequently washed in DPBS.

#### Immunofluorescent staining of isolated myofibers

Isolated myofibers, fixed in 10% formalin and rinsed in DPBS, were subjected to a 1-h block in a blocking solution (10% FBS, 5% Horse Serum (GenDepot) in PBS with 0.2% Triton X-100 (Sigma-Aldrich)) at room temperature. Myofibers were treated with pre-conjugated anti-alpha-tubulin-Alexa fluorophore 488 (catalog number 322588, Invitrogen, [1:250]) and with either an anti-myc-Alexa fluorophore 555 (catalog number 16–225, Millipore Corp., [1:250]) or anti-MAP4-Alexa fluorophore 564 (catalog number sc-390286 AG647, Santa Cruz, [1:250]) antibodies in the blocking solution overnight at 4°C. The following day, myofibers underwent three washes with PBS and were mixed at a 1:1 ratio with the SlowFade TM Glass Antifade Mountant (catalog number S36917, Invitrogen). Myofibers were then mounted onto slides and sealed with a coverslip. After 30-min, slides were imaged under a 40× objective using the Zeiss LSM 880 with Airyscan and laser lines at 488 nm, and either 561 nm or 633 nm. Image analysis and overlay were performed using Fiji-2. To find the correct focal plane for each myofiber (center and surface), we focus first on the bottom and top of the fiber and then set the center as the z-plane exactly between the two extremes. For each image shown (and quantified) 3 stacks (each 1 μm) from the respective myofiber depth were averaged. Image quantification is explained in more detail in Figure S6.

#### Electroporation of constructs in tissue

Mice were placed under anesthesia using 2–5% isoflurane with an oxygen flow rate of 3 L/min using a veterinary anesthesia system. First, the FDB muscle was injected with 0.5 mg/mL hyaluronidase (Sigma). After 2 h, the FDB was injected with 60 μg of plasmid DNA while two electrodes were placed perpendicular to the foot at the top and bottom (approximately 1 cm apart). Electroporation was carried out using the ECM 830 electroporator (Harvard Apparatus) at 90V with a pulse length of 20 ms for 12 pulses at an interval of 1s. After electroporation, mice were returned to clean housing cages where they were allowed to recover. Wet feed was placed in the cage to ensure mice had easy access to food. Mice were left undisturbed (except for regular cage changes) for 5 days. On the 6^th^ day post electroporation, mice were sacrificed, and the FDB muscle was used for analysis.

#### Cloning of Map4 constructs

All constructs in this study were cloned into pcDNA3.1 (Invitrogen) vectors. Different Map4 constructs and pcDNA3.1 backbone regions were amplified from cDNA using Q5 Hot Start High Fidelity Polymerase (New England Biolabs). Both regions were generated such that there was a 15 bp homologous overhang between the backbone and the insert. Fusion of the amplified regions was carried out using the InFusion Snap Assembly Eco Dry kit (Takara Bio).

#### Cell transfections and staining

400,000 LHCN-M2 cells were plated onto 35 mm No. 1.5 Coverslip dishes (MATTEK) and cultured in growth medium at 37°C in 5% CO_2_. The following day, cells underwent transfection with 2 μg of pcDNA vectors expressing myc-tagged MAP4 peptides using Lipofectamine 3000 (catalog number L300001, ThermoFisher) of varying sizes and placed at 37°C in 5% CO_2_. The next day media was replaced, and the cells were placed back at 37°C in 5% CO_2_. The following day (48-h after transfection), cells were rinsed with PBS and fixed in 4% paraformaldehyde. Following fixation, cells were subjected to a 1-h block in a blocking solution (10% FBS, 5% Horse Serum (GenDepot) in PBS with 0.2% Triton X-100 (Sigma-Aldrich)) at room temperature. Cells were treated with pre-conjugated anti-myc-Alexa fluorophore 555 (catalog number 16–225, Millipore Corp., [1:250]) and anti-alpha-tubulin-Alexa fluorophore 488 (catalog number 322588, Invitrogen, [1:250]) antibodies and DAPI (Sigma-Aldrich [1:2000]) in the blocking solution overnight at 4°C. The following day, cells underwent three washes with PBS and were immediately imaged using the Zeiss LSM 880 with Airyscan and laser lines at 405 nm, 488 nm, and 561 nm. Image analysis and overlay were performed using Fiji-2.

#### Tubulin binding of MAP4 isoforms

300 μL of Pierce Protein A/G Magnetic Beads (Catalog number: 8802, ThermoFisher) were washed in PBST and incubated with 90 μg of anti-MAP4 (G-10) catalog number sc-390286, Santa Cruz for 1 h at room temperature to generate antibody-bound-beads. Tubes containing the beads and antibody were placed on a magnetic rack and the supernatant was removed.

6 Gastrocs from WT and *Map4*^ΔE8/ΔE8^ (pooled from 3 mice) were lysed using methods described above. Spun-down lysates and antibody-bound beads were split into 3 equal volumes and incubated together rotating overnight at 4°C. The next day, the tubes were placed on a magnet and the supernatant was removed. Beads and bound proteins were washed 3 times with PBST, each time using the magnet to separate the beads from the supernatant. The beads containing bound proteins were incubated in a 20 μL solution of 10 μM Rhodamine-labeled tubulin (Tubulin protein (rhodamine): porcine brain, catalog number: TL590M-A, Cytoskeleton Inc.) in 1× General tubulin buffer (catalog number: BST01-001, Cytoskeleton Inc) and 1 mM GTP (catalog number: BST06-001, Cytoskeleton Inc) for 30 min as 37°C. After incubation, tubes were placed on a magnet and the supernatant was removed. Beads, bound MAP4 proteins, and bound tubulin were washed 3 times with PBST. The beads were placed in 4× Laemmli Sample Buffer (Bio-Rad) supplemented with 10% 2-Mercaptoethanol and boiled for 10 min to remove all protein from the beads. The beads were run in a gel and transferred for a Western blot of MAP4 (anti-MAP4 (G-10) catalog number sc-390286, Santa Cruz, [1:250]) and imaged under the rhodamine filter for tubulin.

### Quantification and statistical analysis

Experimental design received a statistical test that was relevant to the analysis. Student’s t-test was employed for simple comparisons, while one- or two-way ANOVA was utilized for multi-group comparisons. Each statistical test is outlined in the figure legend of all datasets containing statistical analysis. Power analysis was performed for phenotyping assays so only the mice required were set up for mating prior to experimentation. Molecular experiments usually required a sample number of no less than 3 (*N* ≤ 3). Sample size, statistical test, and definition of error bars is outlined in the figure legend of all graphs. Regardless of the analysis, significance levels were denoted by ∗, ∗∗, ∗∗∗, ∗∗∗∗ for *p* < 0.05, *p* < 0.01, *p* < 0.001, *p* < 0.0001, and 'ns' for not significant.
