# Supplementary material for: Alternatively spliced MAP4 isoforms have key roles in maintaining microtubule organization and skeletal muscle function
Source: iScience. 2024 Oct 5;27(11):111104. doi: 10.1016/j.isci.2024.111104 (PMC11513531; doi:10.1016/j.isci.2024.111104)
Supplement: Document S1. Figures S1–S7 [file mmc1.pdf]

iScience, Volume *27*

Supplemental information

Alternatively spliced MAP4 isoforms have key roles in maintaining microtubule organization and skeletal muscle function

Lathan Lucas, Larissa Nitschke, Brandon Nguyen, James A. Loehr, George G. Rodney, and Thomas A. Cooper

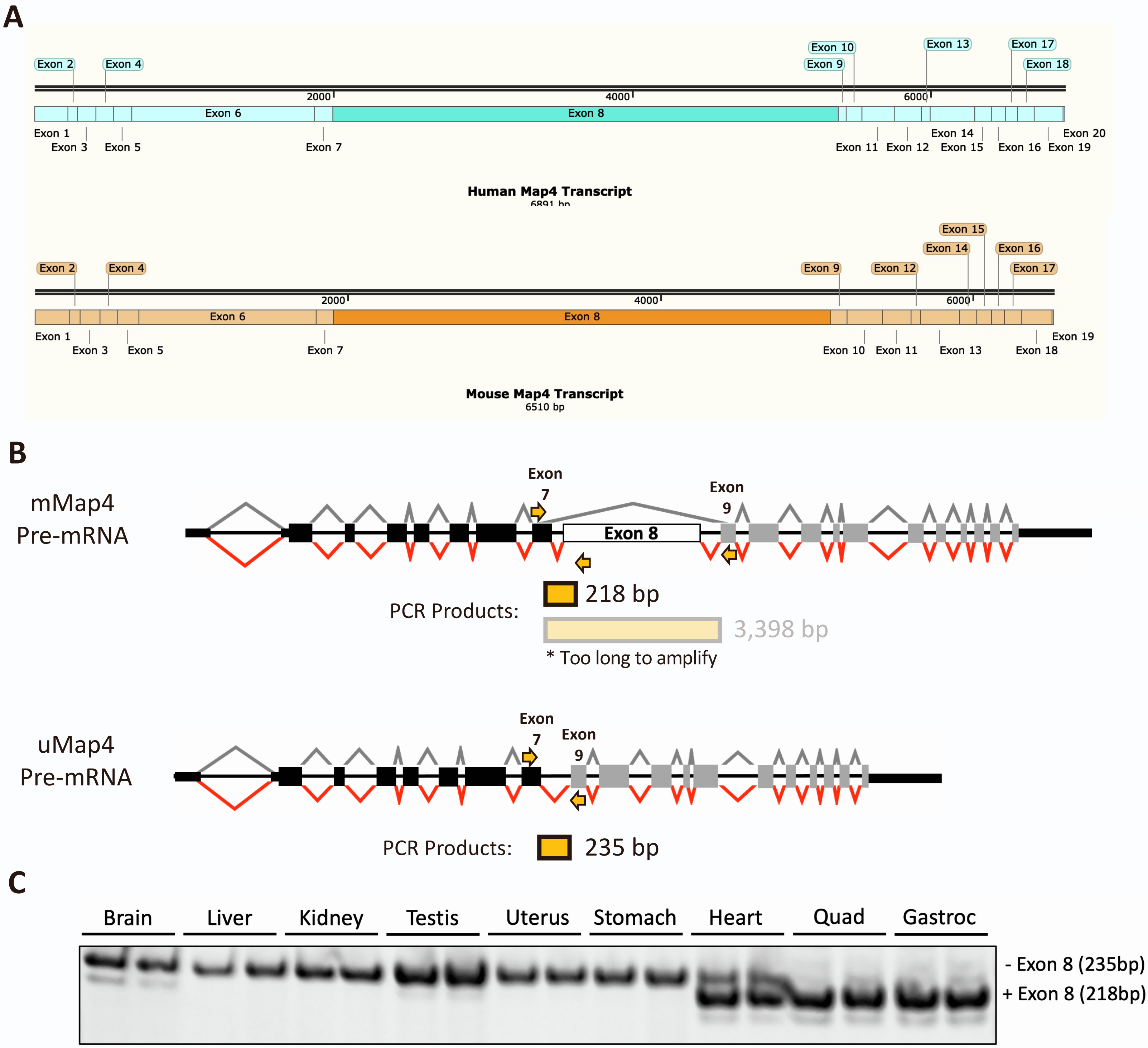

Supplemental Figure 1. (A) Diagrams representing the Map4 mRNA transcripts of human (top) and mouse (bottom) (generated using SnapGene). (B) Primer binding sites (yellow arrows) and PCR product sizes for multiplexed RT-PCR reactions used to simultaneously detect mRNAs that include or exclude exon 8. (C) Representative RT-PCR gel (2 replicates per tissue) showing exon 8 inclusion and exclusion for different mouse tissues.

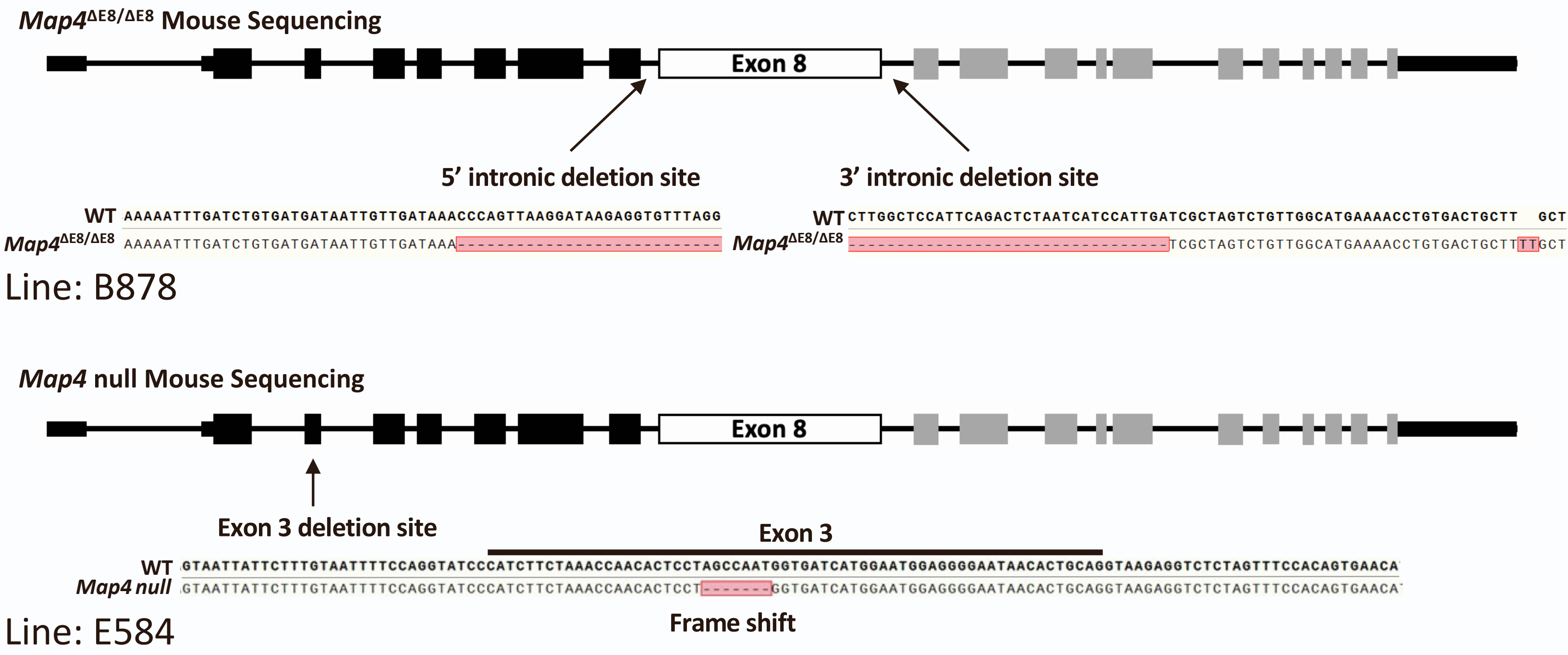

Supplemental Figure 2. Sequencing results to confirm genomic deletions used in this study. The *Map4*$^{\Delta E8/\Delta E8}$ mouse has a deletion of exon 8 (top) while the *Map4* null mouse has a deletion of 7 nucleotides in exon 3 which produces a frameshift and a premature stop codon in exon 4 (bottom).

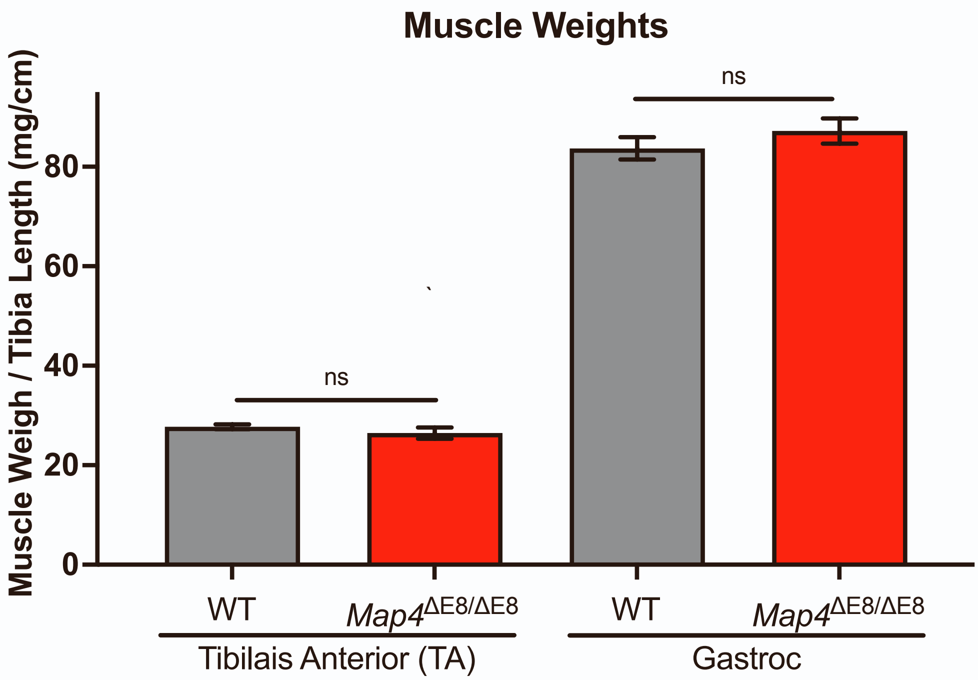

Supplemental Figure 3. Muscle weights of the tibialis anterior and gastrocnemius weights between WT and *Map4*$^{\Delta E8/\Delta E8}$ mice. n = at least 6 mice per genotype. Error Bars = SEM. [ns = not significant]

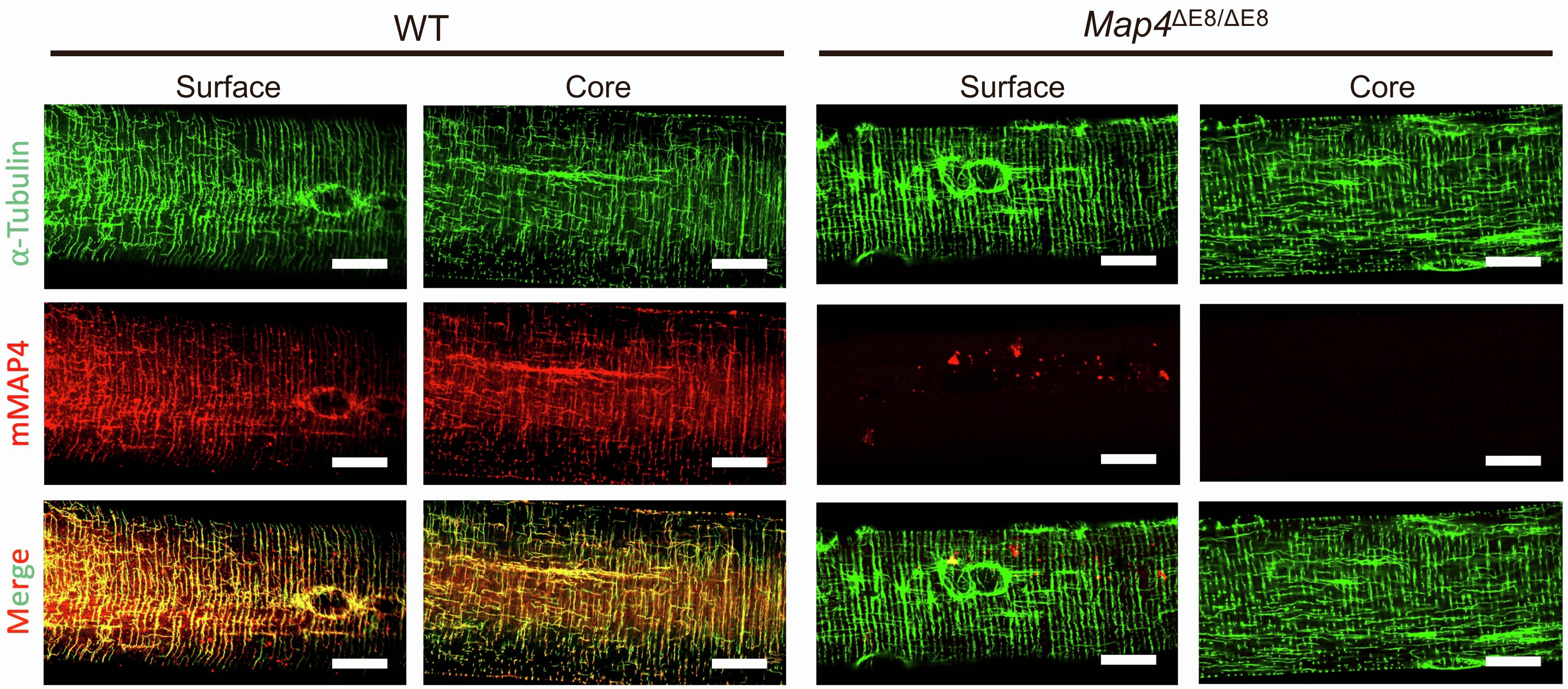

Supplemental Figure 4. Representative immunofluorescent staining at the surface and core of isolated mouse FDB myofibers for alpha-tubulin using an antibody that recognizes the protein region encoded by MAP4 Exon 8 and is therefore specific for mMAP4. Bar, 10µm.

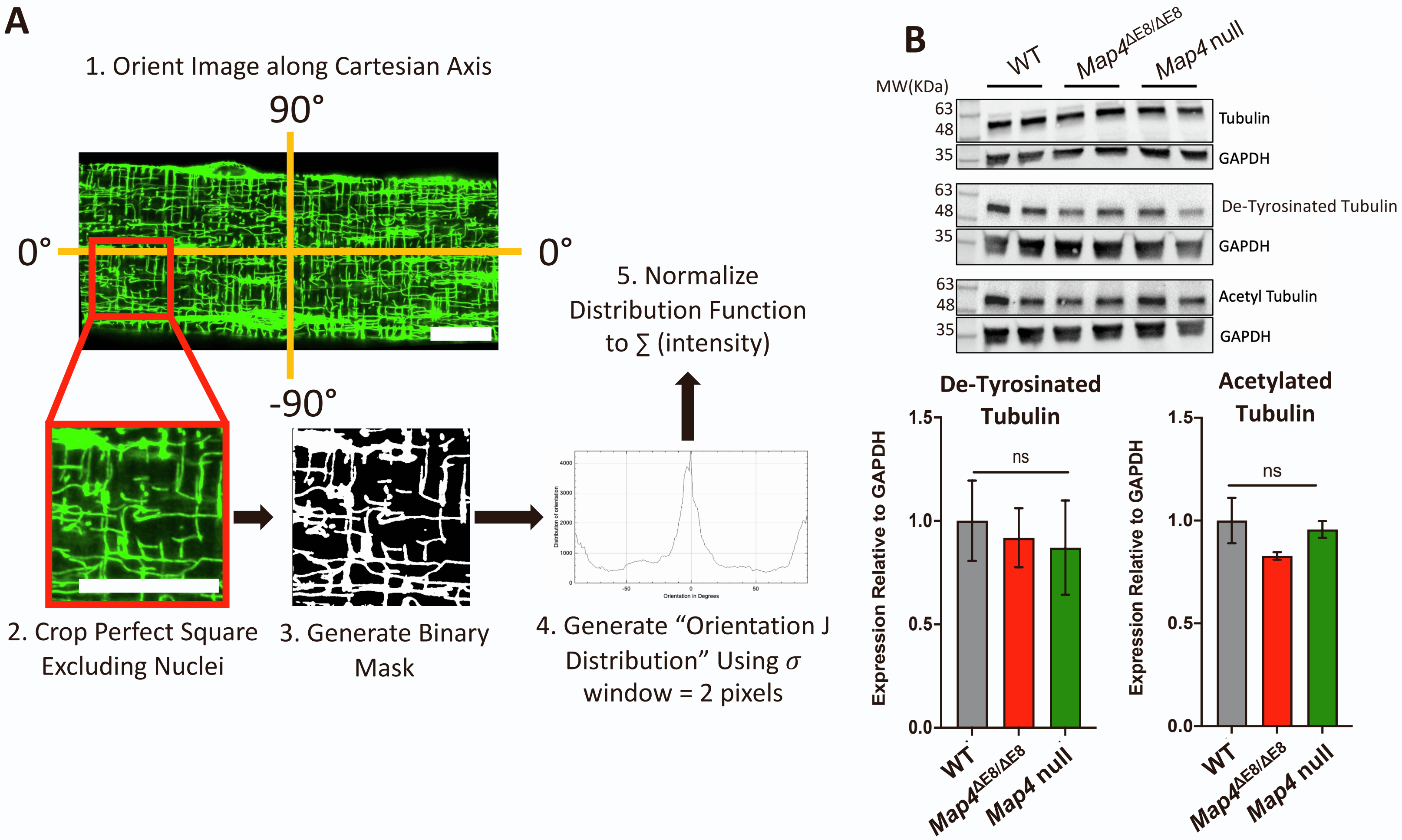

Supplemental Figure 5. (A) Methodology to quantify microtubule organization using a representative image. Bar, 10µm. (B) Western blot (2 replicates per genotype) from gastrocnemius of tubulin and tubulin isoforms used as an indirect measure of microtubule stability within myofibers. n = 2 mice per genotype. Error Bars = SEM. [ns = not significant].

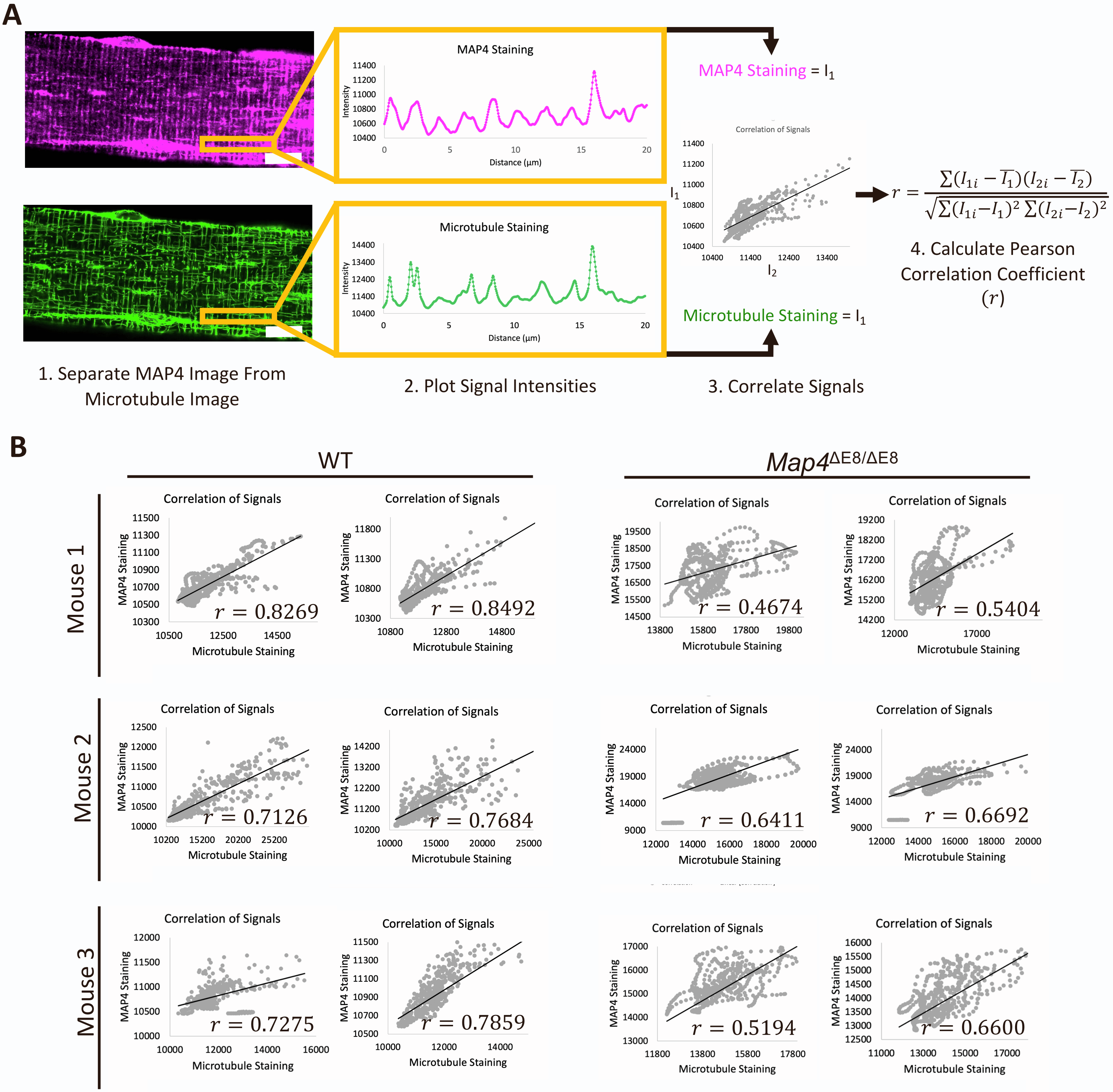

Supplemental Figure 6. (A) Representative methodology of the quantification of co-localization between two immunofluorescent staining images of the same sample. Bar, 10µm. (B) Linear regression plots for all samples used to calculate the Pearson Correlation Coefficient for co-localization of endogenous MAP4 and microtubules. The calculated Pearson correlation value is shown in each plot.

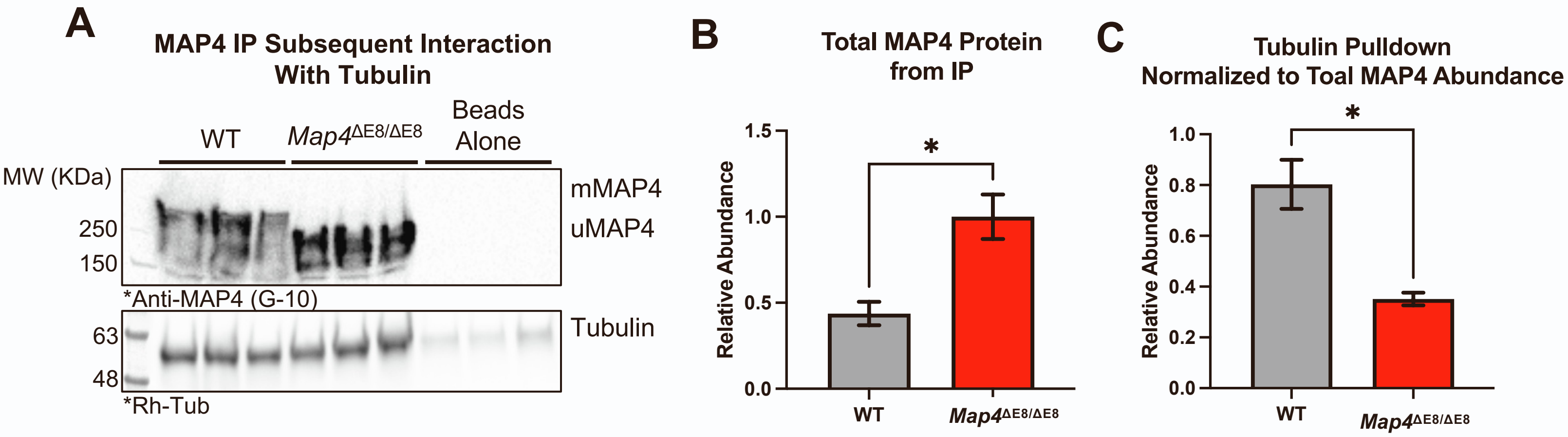

Supplemental Figure 7. Immunoprecipitation of MAP4 and incubation with tubulin indicates mMAP4, compared to uMAP4, has increased interaction with tubulin. A. Western blot and fluorescent (Rhodamine) analysis of a single blot of MAP4 proteins (Anti-MAP4 SG-10) following immunoprecipitation from the quadriceps of WT and *Map4$^{\Delta E8/\Delta E8}$* or from no tissue (beads alone) that were exposed to Rhodamine-labeled tubulin (Rh-Tub) that interacted with the bead-bound MAP4 isoforms. B. Quantification of immunoprecipitated MAP4 abundance statistical comparisons made by one-way ANOVA). n = 3. C. Quantification of Rh-Tub abundance normalized to the amount of immunoprecipitated MAP4 (statistical comparisons made by one-way ANOVA). n = 3. Error Bars = SEM. [*$p<0.05$]
